# Skin and Nail Predictors of Psoriatic Arthritis Development: A Holistic Overview Integrating Epidemiological and Physiopathological Data

**DOI:** 10.3390/jcm13226880

**Published:** 2024-11-15

**Authors:** Zeno Fratton, Ivan Giovannini, Alen Zabotti, Enzo Errichetti

**Affiliations:** 1 Institute of Dermatology, Department of Medicine, University of Udine, 33100 Udine, Italy; 2 Institute of Rheumatology Clinic, “S. Maria della Misericordia” University Hospital, 33100 Udine, Italy

**Keywords:** psoriatic arthritis, nails, predictors, transition, psoriasis

## Abstract

Dermatological manifestations are considered to be of significant importance in identifying individuals with psoriasis at a higher risk of developing arthritis, as rheumatological involvement typically follows the onset of skin/nail lesions. This review summarizes the literature evidence about dermatological predictors of psoriatic arthritis (PsA) development, also analyzing the underlying physiopathological mechanisms and potential biases. Such an integration between statistical evidence and a mechanism-based approach aims to emphasize the most robust skin/nail risk factors upon which clinicians should focus most in daily clinical practice. Accordingly, psoriasis severity and nail changes due to matrix involvement would result in the most relevant risk factors for PsA occurrence, while other possible predictors (e.g., scalp and inverse psoriasis) do not seem to be supported by a significant pathogenetic link.

## 1. Introduction

Psoriatic arthritis (PsA), a complex chronic progressive inflammatory disease that affects the skin, joints, and entheses mostly develops in patients with an established diagnosis of psoriasis (PsO) [1]. Its incidence rises with time after the onset of PsO, reaching up to 20% after 30 years [2], while a recently published meta-analysis has estimated a global prevalence for PsA of 112 per 100,000 adults [3].

The opportunity to prevent and intercept the progression from psoriasis to PsA has garnered the attention of clinicians and researchers, who dedicated their efforts in characterizing the factors more likely to facilitate the transition from psoriasis to PsA [4]. In this context, the wide spectrum of cutaneous findings in patients with psoriasis plays a crucial role in identifying individuals who are at an elevated risk of developing PsA, whether this risk is immediate or extends over the medium to long term [5,6]. Currently available research in the literature indicates that in approximately 75–80% of cases, rheumatological symptoms follow the emergence of skin and nail lesions, highlighting the fundamental role that dermatologists play in the early detection of and intervention in this condition [5,7,8]. However, it is important to note that the existing knowledge regarding dermatological predictors of PsA is predominantly derived from studies conducted within rheumatological settings. This can introduce a selection bias [5], as these studies often prioritize cutaneous findings or lesions manifesting in a more evident way, including those causing significant discomfort to patients, such as those affecting the nails, flexures, or scalp, which are typically easier to identify [5,7]. Additionally, subtle or atypical instances of skin and nail changes that could be clinically significant may go unnoticed by non-dermatologist physicians and certain dermatological lesions may exhibit fluctuating characteristics and may not be observable during study visits. Moreover, many studies have relatively short follow-up periods, complicating the ability to establish a clear link between cutaneous or ungual manifestations and the onset of PsA, as these variables typically precede PsA onset by 7–12 years [5,9,10].

In light of these considerations, the focus of this review will be dedicated to two primary sections: the first section will explore the skin and nail predictors of PsA based on the most reliable studies available in the literature. The second section will investigate the potential pathogenetic connections between dermatological manifestations and the development of PsA. This comprehensive approach aims to integrate statistical evidence with a mechanism-based understanding, thereby emphasizing the most significant skin and nail risk factors that clinicians should prioritize in their daily practice.

## 2. Cutaneous and Nail Predictors Based on the Literature Data

Among the most extensively studied skin predictors for the development of PsA in the medium to long term, the severity of plaque-type psoriasis has gained considerable attention [5]. Several evaluation methods and scores are commonly used in the research and clinical practice to evaluate the extent of cutaneous involvement of individuals affected by psoriasis, namely Psoriasis Area and Severity Index (PASI), Physician Global Assessment (PGA), body surface area (BSA), and involvement of specific areas (i.e., scalp, face, genitals, nails). However, not all scores have shown to exhibit the same predictive value for PsA onset, and even different applications of the same scores have proved to variably correlate with the risk of progression to PsA. For instance, while PASI score may exhibit a weak predictive value for PsA onset when expressed in numerical units [11,12,13], a clear correlation is observed when comparing different classes of severity (see Table 1) [14,15,16].

Specifically, when categorizing severity into three ranges based on PASI scores (<10, 10–20, and >20), a significant higher risk of PsA is evident in patients with PASI scores between 10 and 20, as well as those exceeding 20, compared to individuals with a PASI score below 10. Eder et al. [14] reported a hazard ratio (HR) for these groups of 1.16 and 5.39, respectively [5,14]. Similar results were found in the study by Eder et al. [15] when only two severity classes were considered (PASI < 10 vs. PASI > 10), with an OR of 2.07 (1.47–2.92), while this outcome could not be confirmed in Gisondi et al. [16], who reported a HR of 0.61 (0.29–1.33), although without reaching statistical significance (*p* = 0.216). This trend is further corroborated by analyses comparing severe or moderate/severe psoriasis against mild cases, as defined by the type of therapy administered (e.g., systemic vs. non-systemic treatments) (HR/RR: 1.28–5.02) [17,18,19]. Nonetheless, two studies reported conflicting results in this regard, with comparisons between biologics vs. phototherapy and biologics vs. topical therapy yielding HRs of 1.36 (0.88–2.08) and 2.16 (1.44–3.24), respectively [20], while a comparison between biologics and oral/phototherapy yielded a HR of 4.48 (4.23–4.75) in an analysis by Meer et al. [21]. However, in both studies results were not adjusted for baseline PASI or other markers of severity, hence results should be interpreted with caution as “confounding by indication” and “protopathic bias” might have affected the results [10].

Regarding the localization of psoriasis, a single study with a follow-up period of 13.1 years has demonstrated an association between PsA onset and specific areas of involvement, such as scalp and intergluteal/perianal regions, with HRs of 3.75 and 1.95, respectively [22]. However, these findings have not been replicated in other prospective analyses, with the exception of a study that observed a link between flexural psoriasis and PsA occurrence over a medium- to long-term period exceeding five years, yielding an HR of 2.67 [6]. Notably, no correlation has been established with other cutaneous areas, although patients exhibiting involvement across three or more sites have been identified as having a higher risk of developing PsA, with an HR of 2.24 [22], while only a slightly higher risk (OR 1.18; CI 0.59–2.34) [23] of developing PsA was found when severe psoriasis was categorized as involvement of more than two areas.

**Table 1:** Summary of the main studies reporting the risk of developing PsA correlated to selected skin or nail findings.

Author, Year	Design	Setting	Population	Incident PsA (n)	Follow-Up (Years)	Dermatological Finding	Outcome Measure	Level of Evidence by the Oxford Centre for Evidence-Based Medicine
Eder et al., 2017 [13]	Cohort study	Outpatients Toronto cohort	PsO (n = 410)	57	3.8	Nail pitting	HR 1.98 [0.83–4.74]	2b
Eder et al., 2017 [13]	Cohort study	Outpatients Toronto cohort	PsO (n = 410)	57	3.8	PASI	**HR 1.05 [1.01–1.09]**	2b
Eder et al., 2016 [14]	Cohort study	Outpatients Toronto cohort	PsO (n = 433)	51	4.1	PASI	PASI 10–20 vs. <10 RR 1.16 [0.50–2.64] ^†^ **PASI > 20 vs. <10 RR 5.39 (1.64–17.7)** ^†^	2b
Eder et al., 2016 [14]	Cohort study	Outpatients Toronto cohort	PsO (n = 433)	51	4.1	Nail involvement and lesions	Any psoriatic nail lesion RR 1.36 [0.76–2.45] **Nail pitting RR 2.21 [1.24–3.92]** Nail onycholysis 1.48 [0.83–2.65]	2b
Eder et al., 2012 [15]	Case–control study	Outpatients Toronto cohort	654 cases (PsA) and 401 controls (PsO)	654	N.A.	Severe psoriasis (defined as PASI ≥ 10) based on the maximal PASI in the first 3 years of follow-up	**Severe psoriasis OR 2.07 [1.47–2.92]**	3b
Gisondi et al., 2021 [16]	Cohort study	Outpatients Verona cohort	PsO (n = 464)	51	6.9	Baseline PASI ≥ 10	HR 0.61 [0.29–1.33] ^†^	2b
Gisondi et al., 2021 [16]	Cohort study	Outpatients Verona cohort	PsO (n = 464)	51	6.9	Nail involvement	**HR 3.15 [1.63–6.06]** ^††^	2b
Lewinson et al., 2017 [17]	Cohort study	THIN database	PsO (n = 73,447)	1466	5.1	Psoriasis severity ^†^	**HR** ^††^ **5.02 [4.18–6.04]; *p* < 0.0001**	2c
Egeberg et al., 2018 [18]	Cohort study	Danish data protection database	PsO (n = 10,011)	1269	7.7	Psoriasis severity ^†^ severe vs. mild	**OR 1.28 [1.02–1.61]**	2c
Wilson et al., 2009 [22]	Cohort study	Rochester Epidemiology Project Medical Record	PsO (n = 1593)	57	13.1	No. of affected sites (versus 1)	Unknown HR 1.06 [0.36–3.07] 2 sites HR 0.77 [0.37–1.64] **≥3 sites HR 2.24 [1.23–4.08]**	2b
Wilson et al., 2009 [22]	Cohort study	Rochester Epidemiology Project Medical Record	PsO (n = 1593)	57	13.1	Nail dystrophy (onycholysis, pitting, or hyperkeratosis)	**Nail dystrophy HR 2.24 [1.26–3.98]** ^†^	2b
Thumboo et al., 2002 [23]	Nested case–control	Rochester Epidemiology Project	60 cases and 120 controls	60	N.A.	Nail involvement	OR 1.16 [0.46–2.92]	3b
Thumboo et al., 2002 [23]	Nested case–control	Rochester Epidemiology Project	60 cases and 120 controls	60	N.A.	Generalized PsO at presentation *	OR 1.18 [0.59–2.34]	3b
Ogdie et al., 2021 [24]	Cohort study	THIN database	PsO (n = 8323)	190	4.2	Psoriasis severity evaluated with BSA ^†^	Mild PsO as ref (BSA < 3%) **Moderate PsO (3–10%) HR 1.44 [1.02–2.03]** **Severe PsO (>10%) HR 2.01 [1.19–3.12]**	2c
Belman et al., 2021 [25]	Cohort study	Utah Psoriasis Initiative	PsO (n = 627)	128	7.7	Psoriasis severity (reported by the patient) ^†^	**Untreated Induration HR 1.46 [1.27–1.68]** Untreated desquamation HR 1.22 [1.05–1.42] ^††^ Untreated Erythema HR 1.25 [1.06–1.48] ^††^	2b
Belman et al., 2021 [25]	Cohort study	Utah Psoriasis Initiative	PsO (n = 627)	128	7.7	Psoriasis severity (reported by the investigator at enrollment) ^†^	Induration HR 1.21 [1.02–1.44] ^††^ Desquamation HR 1.16 [0.99–1.37] Erythema HR 1.14 [0.96–1.35]	2b
Belman et al., 2021 [25]	Cohort study	Utah Psoriasis Initiative	PsO (n = 627)	128	7.7	Psoriasis severity	Worst ever BSA HR 1.00 [1.00–1.02]–reported by the patient BSA at enrollment HR 1.00 [1.00–1.02]–reported by investigator	2b
Belman et al., 2021 [25]	Cohort study	Utah Psoriasis Initiative	PsO (n = 627)	128	7.7	Nail involvement	**History of nail involvement HR 2.38 [1.64–3.45]** **Nail involvement HR 2.04 [1.40–2.95]**	2b
Acosta Felquer et al., 2022 [26]	Cohort study	Hospital-based healthcare	PsO (n = 1719)	239	7.3	Nail involvement	**HR 2.7 [1.6–4.5]**	2b

In [14]: ^†^ multivariate model. In [16]: ^†^ Adjusted model for age, sex, scalp, fold and nail localization, psoriasis duration > 10 years, baseline PASI and family history for PsA; ^††^ Multivariate model 2 adjusted for age, sex, scalp, fold and nail localisation, psoriasis duration >10 years, baseline PASI > 10 and family history of PsA. In [17]: ^†^ psoriasis severity was not specified, probably mild vs. moderate-severe; ^††^ multivariable adjusted model for all covariates. In [18]: ^†^ Patients were classified as having severe cutaneous psoriasis if they received systemic therapy for the condition (biological drugs (adalimumab, efalizumab, etanercept, infliximab, or ustekinumab), ciclosporin, psoralen plus ultraviolet A (PUVA), retinoids, or methotrexate) after the onset of psoriasis but before diagnosis of PsA. In [22]: ^†^ multivariate model. In [23]: * skin severity of Ps = was defined as limited (≤2 sites) or generalized (>2 sites) based on involvement of the scalp, elbows, knees, other flexural areas, palms/soles or other sites. In [24]: ^†^ Multivariate Model 2 includes BSA by category (mild, moderate and severe), obesity and depression and adjusts for age and sex. Psoriasis duration was not statistically significant in the multivariable model. In [25]: ^†^ The three components of the investigator global assessment (IGA) analysis (erythema, induration, and desquamation) are on a 0–5 scale with 0 representing phenotype absence and 5 being the most severe. ^††^ not significant after Bonferroni correction. In bold outcome measures that reached statistical significance. N.A. = Not Applicable.

Ogdie et al. [24] studied the impact of psoriasis severity in terms of BSA and compared psoriasis patients having a cutaneous involvement respectively of 3–10% and higher than 10% with those typified by a cutaneous involvement lower than 3%, showing HRs of 1.44 (1.02–2.03) and 2.01 (1.19–3.13), respectively. BSA was evaluated as a marker of disease severity also by Belman et al. [25], but failed to prove an association with PsA onset, yielding HRs of 1.00 (1.00–1.01) and 1.01 (1.00–1.02) for patients reported worst-ever BSA and investigator-reported BSA at enrollment, respectively. In the same study, psoriasis severity was described according to three clinical features reported by patients, i.e., induration, desquamation, and erythema, with a direct correlation with the risk of developing PsA (HRs of 1.46, 1.22, and 1.25, respectively). However, the same severity measures were found to be associated with lower HRs (1.21, 1.16, and 1.14, respectively) and higher *p*-values when reported by investigators at the enrollment.

When examining the clinical subtypes of cutaneous psoriasis, no specific associations with PsA onset have been reported, with the exception of pustular psoriasis (subtype unspecified) [5,6], which has been linked to a heightened risk of transitioning to PsA within one year (HR 3.32) [25] in longitudinal analyses. However, this potential link remains unconfirmed by other studies.

In terms of nail involvement, psoriatic onychodystrophy has not been found to be associated with short-term PsA occurrence (within two years) [5], but a significant association with joint inflammation onset in the medium–long term has emerged [25]. Indeed, psoriatic nail involvement, described as “nail psoriasis” [18], “nail involvement” [23,26], “fingernail psoriasis” [25], or “nail dystrophy” [22], exhibited a strong association with the risk of progression to PsA (HRs ranging between 2.04 and 3.15). Focusing on the types of nail lesions, pitting has been found as the only significant predictor of PsA, with a pooled HR of 2.14 [5]. However, it is noteworthy that pitting was the sole subtype of onychodystrophy assessed in the main studies published in the literature besides onycholysis [13,14], while other psoriatic nail changes were not evaluated [5,8]. We summarize the most essential information regarding cutaneous and nail predictors of PsA in Table 2.

## 3. Pathogenetic Link Between Skin and Nail Predictors and PsA Onset

**Figure 1:**
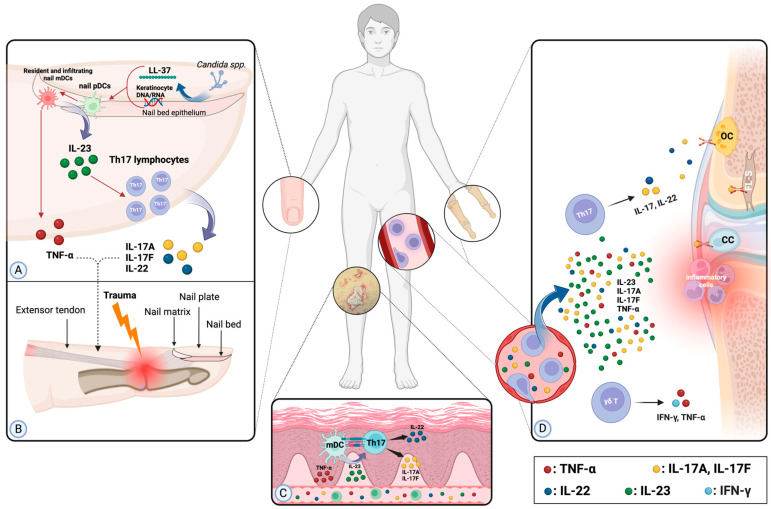
A simplified schematic representation of the possible physiopathological pathway underlying the influence of psoriatic nail matrix and extensive skin inflammation on PsA development. Nail dysbiosis (most commonly *Candida* spp. colonization) induces the production of antimicrobial peptides (LL-37) by the nail bed epithelium, which will bind with DNA/RNA released by nail keratinocytes. LL-37 and DNA/RNA complexes stimulate plasmacytoid dendritic cells (pDCs), with consequent activation of myeloid dendritic cells (mDCs) able to produce interleukin-23, an interleukin regarded as “master regulator” of immunity in the pathogenesis of psoriasis and psoriatic arthritis. IL-23 in turn stimulates Th17 lymphocytes, with subsequent release of downstream effector cytokines (mainly IL-17A, IL-17F, and IL-22 by Th17 lymphocytes and TNF-α by mDCs and stressed keratinocytes) (**Panel A**). Such cytokines reach the near “synovio-entheseal complex” of distal interphalangeal joint, making it more prone to develop inflammation, especially triggered by trauma (resulting from either direct traumatism or mechanical stress transmitted through the nail) (**Panel B**). In psoriatic skin lesions, activated myeloid dendritic cells release several cytokines, especially TNF-α and IL-23, that, in turn, favor the differentiation of Th17 cells producing additional downstream effector cytokines (mainly IL-17A, IL-17F, and IL-22) (**Panel C**). In case of extensive psoriatic involvement, a significant amount of the aforementioned cytokines are released from the skin to the blood stream, thus reaching “synovio-entheseal complex” of distant joints and favoring local inflammation through the activation of resident Th17 cells and gamma-delta T cells, that, in turn, exert their action through the production of cytokines activating effector joint resident cells (such as fibroblast-like synoviocytes, chondrocytes, and osteoclasts, leading to the recruitment of inflammatory cells, stimulation of pro-inflammatory cytokines, and production of matrix metalloproteinases, ultimately resulting in the amplification of inflammation and joint damage) (**Panel D**). (Created with Biorender.com) IL-, interleukin-; TNF-α, tumor necrosis factor α; mDCs, myeloid dendritic cells; pDCs, plasmacytoid dendritic cells; Th17, T helper 17 lymphocyte; γδ cells, gamma-delta cells; IFN-γ, interferon gamma; OC, osteoclast; CC, chondrocyte; FL-S, fibroblast-like synoviocyte; PsA, psoriatic arthritis.

The literature indicates that the severity and extent of psoriasis, along with the involvement of specific areas such as the scalp, flexures, and nails, are primary dermatological predictors of PsA development, particularly in the medium to long term [4,5]. To fully understand the implications of these variables on the risk of arthritis development over time, it is essential to establish the underlying pathogenetic connections. While speculation regarding potential microbial factors exists, the risk associated with scalp and flexural psoriasis in relation to PsA onset currently lacks solid pathophysiological foundations [22]. This uncertainty has led the European League Against Rheumatism (EULAR) to exclude these factors from their recent consensus document on potential PsA risk factors [7]. In contrast, the association between psoriasis severity and nail involvement is supported by a clearer pathogenetic link, which suggests a role in promoting PsA. Figure 1 shows a simplified schematic representation of the physiopathological pathway underlying the possible influence of psoriatic nail matrix and extensive skin inflammation on PsA development.

Specifically, the connection between severe cutaneous involvement and PsA can be attributed to the influence of the main psoriatic cytokines in fostering musculoskeletal inflammation, including interleukin (IL)-23, IL-17, IL-22, and tumor necrosis factor-alpha (TNF-α) [27]. IL-23 is particularly significant in initiating the inflammatory cascade within healthy enthesis by activating resident lymphocyte cells, including innate lymphoid cells and gamma/delta T-cells, which subsequently release downstream effector cytokines [28]. This might find a likely confirmation from the observation that IL-23 inhibitors have shown to possibly prevent the onset of PsA in a case-series including patients with a short-term risk of PsA development [29]. Conversely, IL-17, IL-22, and TNF primarily exert their effects in later stages by activating effector joint resident cells, such as fibroblast-like synoviocytes, chondrocytes, and osteoclasts, leading to the recruitment of inflammatory cells, stimulation of pro-inflammatory cytokines, and production of matrix metalloproteinases, ultimately resulting in the amplification of inflammation and joint damage [27]. Accordingly, elevated systemic levels of these cytokines in patients with severe psoriasis or multisite skin involvement might promote the development of PsA.

While the immunopathogenesis of skin psoriasis has been extensively studied [30], less is known about the immunobiological events at the base of nail psoriasis development, mainly due to an intrinsic difficulty in collecting fresh samples of psoriatic nail unit tissue for immunohistological analysis [31]. However, an interplay of anatomical, microbiological, and immunological factors has emerged as a possible explanation to the connection between nail psoriasis and PsA onset, as will be illustrated in the following lines.

A large body of research in the literature in the last decade has emphasized a higher risk of PsA development at distal interphalangeal joints in patients exhibiting ungual changes, particularly nail pitting [3]. This association can be explained by the close anatomical relationship between the nail and the distal interphalangeal (DIP) joint [32]. Some researchers proposed that the nail should be regarded as a musculoskeletal rather than a skin appendage, as the extensor tendon continues from its bony insertion to envelop the nail root, which contains the nail matrix, while conspicuous bundles of collagen fibers anchor the nail plate to the periosteum. The collateral ligaments form an integrated network on the sides of the joint, anchoring the nail margins. This integrated network connecting the nail plate to the dorsal aspect of the terminal phalanx can be regarded as an enthesis [32,33].

On the microbiological side, psoriasis patients with nail involvement have been shown to carry a higher relative abundance of Candida spp. compared to healthy controls [34,35], which could act as a trigger for exacerbation and maintenance of psoriatic inflammation via induction of antimicrobial peptides production (namely, LL-37 and β-defensins) by epithelial nail bed cells [36] and subsequent activation of the IL-23–Th17 axis cascade [37].

Continuing on the immunological side, as demonstrated by Saulite et al. [38], psoriatic nails appear to express higher amounts of cytokines that have been identified previously in psoriatic skin lesions and that play a role in the pathogenesis of PsA, such as IL-6, IL-8, and TNF-α [39]. Interestingly, the same study revealed higher amounts of IL-10 in psoriatic nails than in the unaffected control group, a cytokine generally designated as anti-inflammatory due to its regulatory and inhibitory properties on pro-inflammatory responses of T lymphocytes [40]. While the presence of IL-10 at the nail unit level is consistent with the established knowledge of the nail apparatus representing a site of relative immune privilege [41], finding a reason for the relative abundance of this cytokine in psoriasis-affected nails poses some challenges. Authors of the study speculate that increased numbers of IL-10 in psoriatic nails might represent some significant differences in the pathogenesis of psoriasis at the cutaneous and nail levels. Higher levels of IL-10 in psoriatic nails might also play a compensatory role against psoriatic inflammation in the nails, in an attempt to maintain the immunomodulatory milieu characteristic for sites of relative immune privilege. Although intriguing, these findings should be taken with caution, as they are derived from the results of a single study in which only tissue specimens from the nail bed were considered, thus failing to provide further clues on the immunobiology of psoriatic nail matrix.

Through the integration of these data, a pathogenetic model can be hypothesized, in which changes in the psoriatic nail (such as nail dysbiosis and consequent production of immunogenic antimicrobial peptides), particularly those involving the psoriatic nail matrix and nail bed, may be considered as a risk factor for PsA development due to the release of pro-inflammatory cytokines (mainly IL-23, IL-17, and TNF) from the nail unit (mainly the matrix) that can spread to the adjacent synovio–entheseal complexes of the digits (especially the distal interphalangeal joint) [22,42], thus becoming sensitized and more prone to the inflammation resulting from trauma (either direct traumatism or mechanical stress transmitted through the nail), the so-called Koebner phenomenon [5,32,43].

Lastly, while pustular psoriasis has been identified as a potential predictor of short-term PsA onset [6], the pathogenetic mechanisms underlying this association remain poorly understood. IL-36 has been identified as the primary cytokine involved in pustular variants of psoriasis [44,45], with recent evidence suggesting its role in driving joint inflammation and diminishing therapeutic response in PsA [46]. This observation aligns with our clinical experience indicating that PsA associated with pustular forms of psoriasis tends to exhibit greater resistance to available treatments.

## 4. Conclusions and Future Perspectives

The integration of statistical evidence and pathophysiological mechanisms emphasizes that the severity of cutaneous psoriasis and nail changes due to matrix involvement are the most relevant risk factors for the development of PsA in the medium to long term, while non-dermatological factors like the detection of arthralgia or an imaging positive for inflammation might represent potential short-term predictors of PsA development [5]. Other potential predictors lack substantial pathogenetic support. Specifically, the release of pro-inflammatory cytokines from extensive psoriatic skin lesions and inflamed nails may sensitize the synovio–enthesal structures (in general and those of the digits, respectively), rendering them more susceptible to inflammation following mechanical stress [5,29].

Despite these insights, significant gaps remain when it comes to the specific roles of various psoriasis subtypes, particularly nail changes, in promoting PsA. Additionally, further research is warranted to explore dermatological predictors in individuals with subclinical PsA [47] as well as the potential development of PsA during psoriasis treatment, necessitating dedicated studies to identify risk factors in this patients subset.

## Figures and Tables

**Table 2:** Summary of the main features of cutaneous and nail predictors of PsA. See the main text and Table 1 for further details.

Field	Predictor	Outcome
Cutaneous	PASI	Studies show a higher risk of developing PsA for higher baseline PASI scores [13,14].
Cutaneous	Psoriasis severity (defined by type of treatment used)	Studies show a higher risk of progression to PsA in patients needing systemic treatment [17,18].
Cutaneous	Extent of cutaneous involvement (generalized vs. localized psoriatic lesions)	Patients with generalized psoriasis have a higher risk of progression to PsA in comparison to patients with localized psoriasis [23].
Cutaneous	BSA	Patients with higher body surface area have a higher risk of progression to PsA [24].
Cutaneous	N. of affected sites	Patients with more than three affected sites show a higher risk of progression to PsA than patients with two or less affected sites [22].
Nail	Nail involvement	Nail involvement predisposes to higher likelihood of progression to PsA [16,23,24,26].
Nail	Nail dystrophy (onycholysis, pitting, or hyperkeratosis)	Nail dystrophy predisposes to higher likelihood of progression to PsA [22].
Nail	Nail pitting	Nail pitting predisposes to higher likelihood of progression to PsA [13,14].

## Data Availability

All data presented are available in this article.

## References

[1] Gladman D.D., Antoni C., Mease P., Clegg D.O., Nash P. (2005). Psoriatic arthritis: Epidemiology, clinical features, course, and outcome. Ann. Rheum. Dis..

[2] Christophers E., Barker J.N.W.N., Griffiths C.E.M., Daudén E., Milligan G., Molta C., Sato R., Boggs R. (2010). The risk of psoriatic arthritis remains constant following initial diagnosis of psoriasis among patients seen in European dermatology clinics. J. Eur. Acad. Dermatol. Venereol..

[3] Lembke S., Macfarlane G.J., Jones G.T. (2024). The worldwide prevalence of psoriatic arthritis—A systematic review and meta-analysis. Rheumatology.

[4] Patt Y.S., Watad A., Giovannini I., Abu-Obeida W.H., Errichetti E., David P., De Marco G., McGonagle D., Zabotti A. (2024). Risk factors for psoriatic arthritis development in psoriasis patients: Myths, pitfalls, and pearls. Clin. Exp. Rheumatol..

[5] Zabotti A., De Lucia O., Sakellariou G., Batticciotto A., Cincinelli G., Giovannini I., Idolazzi L., Maioli G., Tinazzi I., Aletaha D. (2021). Predictors, Risk Factors, and Incidence Rates of Psoriatic Arthritis Development in Psoriasis Patients: A Systematic Literature Review and Meta-Analysis. Rheumatol. Ther..

[6] Eder L., Lee K., Chandran V., Widdifield J., Drucker A., Ritchlin C., Rosen C., Cook R., Gladman D. (2020). The Prediction of Psoriatic Arthritis Tool (PRESTO) Study—Interim Report [abstract]. Arthritis Rheumatol..

[7] Zabotti A., De Marco G., Gossec L., Baraliakos X., Aletaha D., Iagnocco A., Gisondi P., Balint P.V., Bertheussen H., Boehncke W.H. (2023). EULAR points to consider for the definition of clinical and imaging features suspicious for progression from psoriasis to psoriatic arthritis. Ann. Rheum. Dis..

[8] Raposo I., Torres T. (2015). Nail psoriasis as a predictor of the development of psoriatic arthritis. Actas Dermosifiliogr..

[9] De Marco G., Zabotti A., Baraliakos X., Iagnocco A., Aletaha D., Gisondi P., Emmel J., Smolen J.S., McGonagle D.G., Gossec L. (2023). Characterisation of prodromal and very early psoriatic arthritis: A systematic literature review informing a EULAR taskforce. RMD Open.

[10] Errichetti E., Zabotti A. (2024). Biologics in Prevention of Psoriasis to Psoriatic Arthritis Transition: The Need of Prospective Analyses and Stratification According to Time-Related Risk Factors. Dermatol. Ther..

[11] Faustini F., Simon D., Oliveira I., Kleyer A., Haschka J., Englbrecht M., Cavalcante A.R., Kraus S., Tabosa T.P., Figueiredo C. (2016). Subclinical joint inflammation in patients with psoriasis without concomitant psoriatic arthritis: A cross-sectional and longitudinal analysis. Ann. Rheum. Dis..

[12] Elnady B., El Shaarawy N.K., Dawoud N.M., Elkhouly T., Desouky D.E., El Shafey E.N., El Husseiny M.S., Rasker J.J. (2019). Subclinical synovitis and enthesitis in psoriasis patients and controls by ultrasonography in Saudi Arabia; incidence of psoriatic arthritis during two years. Clin. Rheumatol..

[13] Eder L., Polachek A., Rosen C.F., Chandran V., Cook R., Gladman D.D. (2017). The development of psoriatic arthritis in patients with psoriasis is preceded by a period of nonspecific musculoskeletal symptoms: A prospective cohort study. Arthritis Rheumatol..

[14] Eder L., Haddad A., Rosen C.F., Lee K.A., Chandran V., Cook R., Gladman D.D. (2016). The Incidence and risk factors for psoriatic arthritis in patients with psoriasis: A prospective cohort study. Arthritis Rheumatol..

[15] Eder L., Shanmugarajah S., Thavaneswaran A., Chandran V., Rosen C.F., Cook R.J., Gladman D.D. (2012). The association between smoking and the development of psoriatic arthritis among psoriasis patients. Ann. Rheum. Dis..

[16] Gisondi P., Bellinato F., Targher G., Idolazzi L., Girolomoni G. (2022). Biological disease-modifying antirheumatic drugs may mitigate the risk of psoriatic arthritis in patients with chronic plaque psoriasis. Ann. Rheum. Dis..

[17] Lewinson R.T., Vallerand I.A., Lowerison M.W., Parsons L.M., Frolkis A.D., Kaplan G.G., Bulloch A.G.M., Swain M.G., Patten S.B., Barnabe C. (2017). Depression is associated with an increased risk of psoriatic arthritis among patients with psoriasis: A population-based study. J. Investig. Dermatol..

[18] Egeberg A., Skov L., Zachariae C., Gislason G.H., Thyssen J.P., Mallbris L. (2018). Duration of psoriatic skin disease as risk factor for subsequent onset of psoriatic arthritis. Acta Derm. Venereol..

[19] Green A., Shaddick G., Charlton R., Gislason G.H., Thyssen J.P., Mallbris L. (2020). Modifiable risk factors and the development of psoriatic arthritis in people with psoriasis. Br. J. Dermatol..

[20] Watad A., Zabotti A., Patt Y.S., Gendelman O., Dotan A., Ben-Shabat N., Fisher L., McGonagle D., Amital H. (2024). From Psoriasis to Psoriatic Arthritis: Decoding the Impact of Treatment Modalities on the Prevention of Psoriatic Arthritis. Rheumatol. Ther..

[21] Meer E., Merola J.F., Fitzsimmons R., Love T.J., Wang S., Shin D., Chen Y., Xie S., Choi H., Zhang Y. (2022). Does biologic therapy impact the development of PsA among patients with psoriasis?. Ann. Rheum. Dis..

[22] Wilson F.C., Icen M., Crowson C.S., McEvoy M.T., Gabriel S.E., Kremers H.M. (2009). Incidence and clinical predictors of psoriatic arthritis in patients with psoriasis: A population-based study. Arthritis Rheum..

[23] Thumboo J., Uramoto K., Shbeeb M.I., O’Fallon W.M., Crowson C.S., Gibson L.E., Michet C.J., Gabriel S.E. (2002). Risk factors for the development of psoriatic arthritis: A population based nested case control study. J. Rheumatol..

[24] Ogdie A., Shin D.B., Love T.J., Gelfand J.M. (2022). Body surface area affected by psoriasis and the risk for psoriatic arthritis: A prospective population-based cohort study. Rheumatology.

[25] Belman S., Walsh J.A., Carroll C., Milliken M., Haaland B., Duffin K.C., Krueger G.G., Feng B.J. (2021). Psoriasis Characteristics for the Early Detection of Psoriatic Arthritis. J. Rheumatol..

[26] Acosta Felquer M.L., LoGiudice L., Galimberti M.L., Rosa J., Mazzuoccolo L., Soriano E.R. (2022). Treating the skin with biologics in patients with psoriasis decreases the incidence of psoriatic arthritis. Ann. Rheum. Dis..

[27] Lee B.W., Moon S.J. (2023). Inflammatory Cytokines in Psoriatic Arthritis: Understanding Pathogenesis and Implications for Treatment. Int. J. Mol. Sci..

[28] Watad A., Cuthbert R.J., Amital H., McGonagle D. (2018). Enthesitis: Much More Than Focal Insertion Point Inflammation. Curr. Rheumatol. Rep..

[29] Zabotti A., Giovannini I., McGonagle D., De Vita S., Stinco G., Errichetti E. (2022). Arthritis Interception in Patients with Psoriasis Treated with Guselkumab. Dermatol. Ther..

[30] Sieminska I., Pieniawska M., Grzywa T.M. (2024). The Immunology of Psoriasis-Current Concepts in Pathogenesis. Clin. Rev. Allergy Immunol..

[31] Ventura A., Mazzeo M., Gaziano R., Galluzzo M., Bianchi L., Campione E. (2017). New insight into the pathogenesis of nail psoriasis and overview of treatment strategies. Drug Des. Devel Ther..

[32] McGonagle D., Tan A.L., Benjamin M. (2009). The nail as a musculoskeletal appendage—Implications for an improved understanding of the link between psoriasis and arthritis. Dermatology.

[33] Tan A.L., Benjamin M., Toumi H., Grainger A.J., Tanner S.F., Emery P., McGonagle D. (2007). The relationship between the extensor tendon enthesis and the nail in distal interphalangeal joint disease in psoriatic arthritis—A high-resolution MRI and histological study. Rheumatology.

[34] Wang S., Wang R., Song Y., Wan Z., Chen W., Li H., Li R. (2022). Dysbiosis of nail microbiome in patients with psoriasis. Exp. Dermatol..

[35] Wang S., Song Y., Wan Z., Chen W., Wang R., Li R. (2022). Characterisation of the nail microbiome in psoriatic and nonpsoriatic patients with onychomycosis. Mycoses.

[36] Saulite I., Pilmane M., Kisis J. (2017). Expression of antimicrobial peptides in nail psoriasis and normal nails. Acta Derm. Venereol..

[37] Ji C., Wang H., Bao C., Zhang L., Ruan S., Zhang J., Gong T., Cheng B. (2021). Challenge of nail psoriasis: An update review. Clin. Rev. Allergy Immunol..

[38] Saulite I., Pilmane M., Guenova E., Kisis J. (2017). Expression of inflammatory cytokines in psoriatic nails. J. Eur. Acad. Dermatol. Venereol..

[39] Veale D.J., Fearon U. (2018). The pathogenesis of psoriatic arthritis. Lancet.

[40] Baliwag J., Barnes D.H., Johnston A. (2015). Cytokines in psoriasis. Cytokine.

[41] Ito T., Ito N., Saathoff M., Stampachiacchiere B., Bettermann A., Bulfone-Paus S., Takigawa M., Nickoloff B.J., Paus R. (2005). Immunology of the human nail apparatus: The nail matrix is a site of relative immune privilege. J. Investig. Dermatol..

[42] McGonagle D. (2009). Enthesitis: An autoinflammatory lesion linking nail and joint involvement in psoriatic disease. J. Eur. Acad. Dermatol. Venereol..

[43] McGonagle D., Benjamin M., Tan A.L. (2009). The pathogenesis of psoriatic arthritis and associated nail disease: Not autoimmune after all?. Curr. Opin. Rheumatol..

[44] Misiak-Galazka M., Zozula J., Rudnicka L. (2020). Palmoplantar Pustulosis: Recent Advances in Etiopathogenesis and Emerging Treatments. Am. J. Clin. Dermatol..

[45] Manome-Zenke Y., Ohara Y., Fukui S., Kobayashi D., Sugiura K., Ikeda S., Arai S. (2022). Characteristics of Patients with Generalized Pustular Psoriasis and Psoriatic Arthritis: A Retrospective Cohort Study. Acta Derm. Venereol..

[46] Boutet M.A., Nerviani A., Lliso-Ribera G., Lucchesi D., Prediletto E., Ghirardi G.M., Goldmann K., Lewis M., Pitzalis C. (2020). Interleukin-36 family dysregulation drives joint inflammation and therapy response in psoriatic arthritis. Rheumatology.

[47] Zabotti A., Fagni F., Gossec L., Giovannini I., Sticherling M., Tullio A., Baraliakos X., De Marco G., De Vita S., Errichetti E. (2024). Risk of developing psoriatic arthritis in psoriasis cohorts with arthralgia: Exploring the subclinical psoriatic arthritis stage. RMD Open.

